# Acute effect of moderate and high-intensity interval exercises on asprosin and BDNF levels in inactive normal weight and obese individuals

**DOI:** 10.1038/s41598-023-34278-6

**Published:** 2023-04-29

**Authors:** Halil İbrahim Ceylan, Mehmet Ertuğrul Öztürk, Deniz Öztürk, Ana Filipa Silva, Mevlüt Albayrak, Özcan Saygın, Özgür Eken, Filipe Manuel Clemente, Hadi Nobari

**Affiliations:** 1 Physical Education and Sports Teaching Department, Kazim Karabekir Faculty of Education, Ataturk University, Erzurum, Turkey; 2 Vocational School of Health Services, Ataturk University, Erzurum, Turkey; 3 Escola Superior Desporto e Lazer, Instituto Politécnico de Viana do Castelo, Rua Escola Industrial e Comercial de Nun’Álvares, 4900-347 Viana do Castelo, Portugal; 4 The Research Centre in Sports Sciences, Health Sciences and Human Development (CIDESD), 5001-801 Vila Real, Portugal; 5 Research Center in Sports Performance, Recreation, Innovation and Technology (SPRINT), 4960-320 Melgaço, Portugal; 6 Coaching Science, Faculty of Sports Sciences, Mugla Sitki Kocman University, Muğla, Turkey; 7 Department of Physical Education and Sport Teaching, Faculty of Sports Sciences, Inonu University, Malatya, Turkey; 8 Instituto de Telecomunicações, Delegação da Covilhã, 1049-001 Lisbon, Portugal; 9 Department of Exercise Physiology, Faculty of Educational Sciences and Psychology, University of Mohaghegh Ardabili, Ardabil, 56199-11367 Iran; 10 Faculty of Sport Sciences, University of Extremadura, 10003 Cáceres, Spain

**Keywords:** Feeding behaviour, Metabolic diseases, Obesity, Hormones

## Abstract

This study aimed to examine the acute effects of moderate-intensity aerobic and high-intensity interval exercise protocols on Asprosin and Brain-Derived Neurotrophic Factor (BDNF) levels in inactive normal weight and obese individuals. A total of 20 male individuals aged 18–65 years, ten normal weight (NW) (Body Mass Index (BMI): 18.5–24.99 kg/m^2^) and 10 obese (Ob) (BMI: 24.99–35.00 kg/m^2^) participated in this study, voluntarily. Moderate aerobic exercise (AE) (main circuit 30 min, between 40 and 59% of Heart Rate Reserve: HRR) and High-Intensity Interval exercise (HIIE) running protocols (main circuit 20 min, between 75 and 90% of the HRR for 1 min*10 times, and 1-min active rest at 30% of the HRR) was applied to the volunteer participants in the morning hours (08.00–10.00 a.m.), following the night fasting (at least 8–10 h) for at least 3 days between each other. Blood samples were collected from the participants before and immediately after each exercise protocol, and serum asprosin and BDNF hormone levels were determined by Enzyme-Linked Immunosorbent Assay” method. Basal serum asprosin was found to be significantly higher in the Ob group compared to the NW group (*p* < .001), while the basal serum BDNF hormone was found to be lower (*p* < 0.05). It was observed that the serum asprosin level of both groups decreased significantly after both AE and HIIE protocols (*p* < 0.05). In addition, there was a significantly higher decrease in serum asprosin level in the Ob group compared to the NW group after HIIE protocol. For the Ob group, serum BDNF level increased considerably after HIIE protocol compared to AE protocol (*p* < 0.05). Serum asprosin was found to be higher in the Ob group, while the serum BDNF was found to be lower. In addition, the acute exercises of different intensity significantly affected hormones that regulate appetite metabolism. In particular, it was observed that the HIIE protocol had a greater effect on the regulation of appetite (hunger-satiety) in the Ob group. This result can be taken into account when planning training programs for these individuals.

## Introduction

Physical exercise is one of the most common non-pharmacological approaches to preventing non-communicable diseases, such as obesity, cardiovascular diseases, 13 types of cancer, type 2 diabetes mellitus, and chronic respiratory diseases while improving health and well-being^1–3^. Physical exercise represents a structured physical activity in which people are subjected to a given locomotor and mechanical stimulus that will result in a specific physiological and biochemical response^4^. The physiological and biochemical response aims to adjust the organism to the demands imposed by the physical stimulation of exercise^5^. This is the meaning of acute response to the exercise. Interestingly, the acute responses impact different organic systems since a cascade of events occurs pending on the type, mode, duration, and intensity of exercise^6^.

For example, exercise seems to stimulate the release of neurotransmitters and neurotrophins in an activity-dependent manner^7^. Based on that, exercise can acutely induce a cascade of events in the brain, namely the ones related to brain plasticity^8^. In this specific case, the brain-derived neurotrophic factor (BDNF) is one of the critical mechanisms underlying exercise-induced brain plasticity and cognitive function since it is suggested that BDNF mediates the impact of exercise on cognition and mood^9,10^. The BDNF is a protein found in the hippocampus, cerebral cortex, hypothalamus, and cerebellum brain regions. BDNF has been associated with neural development and functioning, specifically with the cases of neurogenesis, dendritic growth, and the long-term potential of neurons^11^. Higher BDNF levels are associated with better spatial, episodic recognition and memorization^9^. Besides that, it is also associated with better hippocampal functioning^9^.

The World Health Organization^12^ and the American College of Sports Medicine (ACSM)^13^ recommend at least 150 min of moderate-intensity aerobic physical activity (40–60% of VO2max) per week or 75 min of high-intensity aerobic physical activity (60–85% of VO2max) to maintain health and improve aerobic fitness levels for obese and healthy adults aged 18–65 years. In recent years, high-intensity interval exercise (HIIE) is estimated to be the most popular trend in fitness, according to the ACSM Annual Fitness Trend Forecast^14^. HIIE is characterized by repeated short-term explosive-intensity anaerobic activities (≥ 85–90% VO2max for health subjects or ≥ 80% VO2max for clinical populations such as obesity), interspersed by periods of passive recovery or low-intensity exercise for recover, and it typically takes less than 30 min^6,15^. Recent studies have recognized that HIIE versus traditional moderate-intensity continuous aerobic exercise, particularly in overweight and obese adults, has been demonstrated to be a time-efficient exercise strategy to further improve waist circumference, body fat percentage, aerobic fitness, and cardio-metabolic health in a shorter time^16^. In addition, in previous studies, it has been observed that compared to aerobic exercise protocol, HIIE protocols reduce the subjective feeling of hunger, and cause an increase in appetite related hormones such as glucagon-like peptide 1, which is the hormone that creates a sense of satiety^17^, and a decrease in the level of ghrelin, which is the hormone that triggers the feeling of hunger^18^.

In literature, HIIE^19^, has been shown to have a positive effect on BDNF^20^, which is a protein that is critical for the growth and survival of neurons in the brain. Studies have shown that HIIE can increase the levels of BDNF in the brain, leading to improved cognitive function, enhanced mood, and reduced risk of neurological disorders^21^. One way that HIIE may increase BDNF levels is through its ability to stimulate the production of growth factors such as insulin-like growth factor 1 (IGF-1) and vascular endothelial growth factor (VEGF)^22^. These growth factors have been shown to promote the growth of new blood vessels and neurons in the brain, leading to improved cognitive function and reduced risk of neurological disorders^23^. Additionally, HIIE has been shown to increase the production of hormones such as cortisol^24^ and adrenaline^25^, which are known to stimulate the production of BDNF. This may help explain why HIIE protocol has been found to have such a powerful effect on BDNF levels compared to moderate intensity aerobic exercise protocol^26^.

The relationship between BDNF levels and obesity have been explored in obese and overweight humans^27^. In addition to the effect of BDNF on brain health and cognitive function mentioned above, recent studies have shown that BDNF actively controls food intake and regulates body weight at the hypothalamic level^28,29^. According to these studies, it has been determined that BDNF-producing neurons in the paraventricular hypothalamus reduce food intake and act as an anorexigenic factor, creating a feeling of satiety. Another studies have reported that mutations in the BDNF gene and its receptor TrkB increase food intake in mice and humans, leading to the development of severe obesity^30,31^. In literature, previous study in humans have shown that individuals with lower levels of BDNF may be at a higher risk for obesity^32^. For example, one study found that obese individuals had lower levels of BDNF compared to normal weight individuals^33^. In contrast, in a different study, it was stated that there was no significant difference in terms of basal BDNF levels between obese and normal weight individuals^27^. However, it's important to note that the relationship between BDNF and obesity is complex and not fully understood.

The additional effects beneficial of HIIE protocol on obese or overweight populations can also be in the regulation of asprosin levels^34^. Asprosin is a hormone that was discovered in 2016 and is primarily produced by white adipose tissue (fat cells)^35^. It is a small protein that is involved in the regulation of glucose homeostasis (the balance of glucose in the body) and energy metabolism^36^. Moreover, asprosin is a centrally acting orexigenic hormone that triggers the feeling of appetite just as grelin hormone. Also, it regulates the liver to release hepatic glucose and increase plasma glucose levels while helping to control appetite^36^.

Asprosin has been found to be elevated in conditions such as obesity, type 2 diabetes, and insulin resistance, suggesting that it may play a role in the development of these metabolic disorders^37,38^. Exercise has been shown to have a beneficial impact on asprosin levels^39^. Specifically, HIIE protocols has been found to decrease asprosin levels in obese adults. In a comparison between healthy and obese adults^40^, a 30-min aerobic exercise seems to decrease asprosin levels in both morning and evening, despite overweight and obese showed more decreases in the asprosin level after training. In anaerobic exercise observed, a decline of asprosin after 3-min of exercise while augmenting within 60 min after the training^41^.

In the light of the above considerations, asprosin (increases the feeling of hunger) and BDNF (creates a feeling of satiety) are current hormones associated with appetite metabolism, which constitute a large part of the physiology of obesity. Taking into account the aforementioned studies, it has been observed that the asprosin is high in obese individuals, whereas the BDNF is low. Overall, the relationship between BDNF, asprosin, and HIIE exercise appears to be complex, with different types and intensities of exercise having varying effects on asprosin levels. However, exercise does seem to have a beneficial impact on BDNF and asprosin levels in obese and overweight individuals, which may contribute to the metabolic improvements associated with exercise. Given the limited research dedicated to analyzing the impact of HIIE on BDNF and asprosin levels in obese and overweight individuals, this study aims to examine the effects of moderate and high-intensity exercise on BDNF and asprosin levels in both healthy and obese people. Based on the available evidence, we hypothesize that there will be a difference in basal asprosin and BDNF hormone levels between individuals of normal weight and those who are obese. Additionally, we expect that the HIIE protocol will have a greater impact on these hormones compared to the AE protocol, and this effect will be more pronounced in the obese group.

## Method

### Participants

A total of 20 adult men who reported that they did not engage in regular exercise or physical activity participated in this study voluntarily. Participants were divided into two groups as 10 normal weight (NW: BMI: 18.5–24.99 kg/m^2^) and 10 obese (Ob: BMI: 30–39.9 kg/m^2^) based on the World Health Organization/International Association for the Study of Obesity/International Obesity Task Force definition of obesity^42^. Moreover, according to the American Exercise Council, a body fat (%) between 13 and 21% for men is considered "healthy", while over 25% is considered obese^43^. Additionally, a previous meta-analysis study has reported a cut-off point of over 25% body fat (%) as a threshold for defining obesity^44^. Therefore, in our study, both BMI and body fat (%) values of the participants have been taken into account for creating both groups (NW and Ob). The sample group of the present study study determined by using the criterion sampling method, which is one of the purposive sampling methods^45^. This method requires specific criteria to be met for individuals to be included in the study. Accordingly, face-to-face interviews were conducted with potential participants prior to the study. The participants were selected based on the inclusion criteria stated below; healthy adult male, 18–65 years, physically inactive; not participating in < 150 min/week moderate intensity or < 75 min/week vigorous intensity, or an equivalent combination of the two diffrent intensities^46^. According to the latest data from the World Health Organization^47^, it has been stated that more than 1.9 billion adults who are 18 years and older are overweight (aged 18–65 years) worldwide, and of those, over 650 million are considered to be obese. Based on these data, the present study was conducted on the adult population aged 18–65 years to contribute to understanding the pathophysiology of obesity in adults. Exclusion criteria for all groups were: participants with any cardiovascular disease, metabolic diseases such as diabetes or hypertension, participants who use cigarettes, drugs and alcohol, participants whose BMI is below 18.5 for NW group or over 40 kg/m^2^ for the Ob group. All participants were adequately informed about the purpose, content, method (exercise protocols and functions of hormones) and possible risk factors related to the experimental design. Afterwards, the participants signed the Informed Consent Form. The present study was conducted in accordance with the principles stated in the Declaration of Helsinki. In addition, the present study was approved by Atatürk University Faculty of Medicine Clinical Research Ethics Committee (Meeting Number: 08, Decision no: 54, Date: 26.12.2019).

### Sample size

The minimum sample size for the present study was calculated using G-power software 3.1.9.7. (University of Dusseldorf, Dusseldorf, Germany)^48^. According to this analysis; a priori and F tests were used to calculate power following our study's design; ANOVA: repeated measurements, within-interaction analysis; α err prob = 0.05; minimum effect size = 0.35^38,49^; the number of groups = 2; the number of measurements = 2; and power (1-β err prob) = 0.80. Based on repeated analysis of variance measures, the minimum sample size for statistical significance was a total of 20 participants with an actual power of 84.1%.

### Experimental design

**Figure 1:**
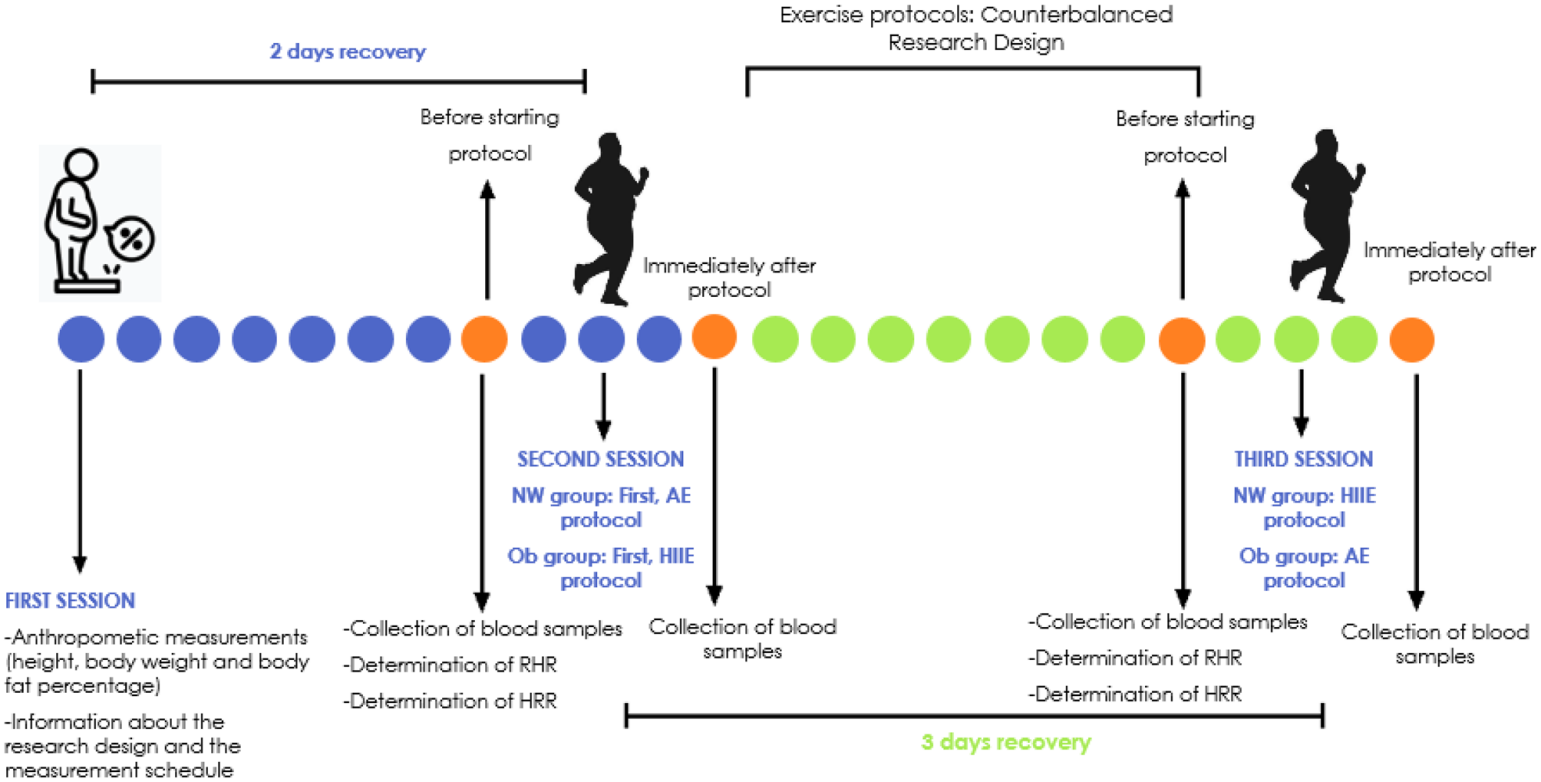
Experimental design of the study.

The experimental design of the present study is shown in Fig. 1. In our study, the counter-balanced research design was used to minimize the sequencing effect in administering exercise protocols to participants^50^ (For NW group = second session: AE protocol, third session: HIIE protocol, for Ob group: second session: HIIE protocol, third session: AE protocol). Measurements of the study were completed in about 7 days in 3 sessions. In the first session, all participants' height, body weight and body fat percentage were measured. Then, the participants in each group participated in the AE or HIIE running protocols in a counterbalanced manner. Before starting the protocols, each participant was worn a Polar watch (V800) to ensure that they remained within the desired HRR (calculated by the researchers) during the protocols. Also, they were verbally motivated throughout the practice to perform the exercises at the targeted heart rate. In the second and third sessions, the participants' resting heart rate (RHR) was determined. Then, using the formula developed by Karvonen et al.^51^, HRR was calculated for both AE and HIIE protocols, and exercise intensity was determined. The interval between the second and third sessions was determined at least 3 days^38,52^. Both exercise protocols were carried out between 08.00 and 10.00 a.m. hours following the night fasting (at least 8–10 h). The morning exercises are performed on an empty stomach (before breakfast) because the activities on an empty stomach trigger more fat burning, that is, beta-oxidation, than the exercises performed on a full stomach. Previous studies showed that morning exercises on an empty stomach had a greater effect on hormones regulating appetite regulation^53,54^. Blood samples were collected from the participants before and immediately after AE and HIIE protocols^33,49^, and serum asprosin and BDNF levels were determined. Moreover, the participants were asked to sleep for at least eight hours and to avoid excessive fat intake; high-intensity exercise outside of their daily routines; and caffeine, stimulants, alcohol, and nutritional supplements (vitamins and antioxidants) before both exercise protocols^49,55–57^.

### Aerobic and high-intensity interval exercise protocol (AE and HIIE)

In literature, it is recommended that overweight and obese individuals should perform moderate-intensity aerobic exercise for 30–60 min, 5 days a week, between 40 and 59% of their heart rate reserve (HRR). In addition, it was reported that exercise performed at 60% or more of HRR provided additional health benefits^13^.

In our study, The AE protocol lasted approximately 50 min, including 10 warm-ups, main circuit: 30 min of exercise, 10 min of cool-down. A 30-min moderate-intensity AE running exercise protocol was applied to all participants in the range of 40%-59% of the HRR recommended by the American College of Sports Medicine^13^ for NW and Ob adult individuals. After the exercise, the session was completed with 10 min cool-down exercises.

HIIE running protocol (approximately 35 min in total; 3 min’ warm-up, main circuit: 20 min of exercise, 10 min of cool-down) were used in the previous studies conducted by Saucedo Marquez et al.^58^ and Matos et al.^17^. According to this protocol; the participants warmed up at 30% of their 3-min HRR with jogging. After warm up, they ran for 1 min between 75 and 90% of their HRR. Afterwards, the participants were given a 1-min rest. This protocol was repeated 10 times in the form of 1 min of loading in the range of 75%-90% of the HRR, and 1 min of rest (light jogging at 30% of the HRR). The changes in heart rate in the exercise protocols of the participants were monitored with a Polar watch (V800).

### Anthropometric measurements

In our study, body mass was determined using a body composition monitor with an accuracy of 0.1 kg (Tanita SC-330S, Amsterdam, Netherland), and height was measured using a stadiometer with an accuracy of 0.1 cm (Seca 217, Hamburg, Germany). Before body mass and body fat measurements, participants were informed in detail about the criteria recommended by the World Health Organization^59^ to minimize measurement errors, and they followed the specified instructions. Moreover, body mass index (BMI) was calculated by dividing body mass by the square of the height (kg/m^2^).

### Blood collection and analysis methods

Before and after AE and HIIE protocols were performed in the morning hours (08.00–10.00 a.m.), 2 ml blood samples were taken from the antecubital vein of each participant before and immediately after both exercise protocols^33,49^, and placed in biochemistry tubes with EDTA. Blood samples were incubated at room temperature for 30 min and then centrifuged at 3000 rpm for 15 min. Afterwards, they were transferred to two microcentrifuge tubes containing approximately 100 µl of serum, each via automatic pipettes, and the serum samples were stored in a deep freezer at − 80 °C until analysis. Serum asprosin (Human Asprosin ELISA Kit, Cat No: E4095Hu, Shanghai, China, Intra assay: CV < 8%, Inter-assay: CV < 10%, sensitivity: 0.023 ng/mL, standard curve range: 0.5–100 ng/mL) and serum BDNF (Human BDNF ELISA Kit, Bioassay Technology Laboratory, catalog number 14: E1302Hu; intra assay: CV < 10, precision; 0.23 ng/mL, standard curve range: 0.05–10 ng/mL) levels were measured by the expert team involved in the study using the Biotech Epoch Microplate Spectrophotometer brand ELISA device with the “Enzyme-Linked Immunosorbent Assay” (ELISA) method under the working procedures specified in the catalogs of the manufacturers. Both hormone levels were calculated according to the calibration curve, and serum amounts were determined.

### Statistical analysis

The data analysis was conducted in the SPSS 26.0 (SPSS, Inc., Chicago, IL, USA) program. Serum asprosin and BDNF values of NW and Ob groups before and after AE and HIIE were shown in bar charts as mean ± standard deviation, and individual variations were shown in scatter plots. Whether the data showed a normal distribution was determined by examining the Shapiro–Wilk test results, skewness, and kurtosis values. According to these results, it was seen that the data had a normal distribution. Independent Sample t Test was used to compare mean values of serum asprosin and BDNF (basal) in terms of groups (NW and Ob). Moreover, the Receiver Operating Characteristic Analysis test was performed to determine the predictive diagnostic value of serum levels of asprosin and BDNF in obesity. The comparison of mean serum asprosin and BDNF values in different BMI groups (NW and Ob) according to different exercise intensities (AE and HIIE) was performed with Two-way Repeated Measures ANOVA with a 2 × 2 design (Groups*Pre-test-Post test). In cases of significant difference, pairwise comparisons were analyzed with the Bonferroni test. Moreover. the Cohen’s D effect-size (ES) was performed to determine the effect magnitude through the difference of two means divided by the standard deviation from the data, and the following criteria were used: < 0.2 = trivial, 0.2 to 0.6 = small effect, 0.6 to 1.2 = moderate effect, 1.2 to 2.0 = large effect, and > 2.0 = very large^60^. The significance level was accepted as *p* < 0.05.

### Ethical approval and consent to participate

The study was conducted according to the Declaration of Helsinki guidelines and was approved by the Ataturk University Faculty of Medicine Clinical Research Ethics Committee (Meeting Number: 08, Decision no: 54, Date: 26.12.2019). Informed consent was obtained from all subjects agreed to participate in this study and answered the questionnaire.

## Results

As shown in Table 1, the age, BMI, and body fat (%) values of the NW group were as 39.9 ± 4.58 years, 24.02 ± 0.68 kg/m2, and 20.03 ± 1.01, respectively. In the Ob group, the age, BMI and body fat (%) values were as 40.3 ± 3.88 years, 32.77 ± 1.72 kg/m2 and 33.97 ± 1.61, respectively.

**Table 1:** The age, BMI and body fat (%) values of the participants.

Variables	Groups	
	NW M ± S.D	Ob M ± S.D
Age (years)	39.9 ± 4.58	40.3 ± 3.88
BMI (kg/m^2^)	24.02 ± .68	32.77 ± 1.72
Body Fat (%)	20.03 ± 1.01	33.97 ± 1.61

**Figure 2:**
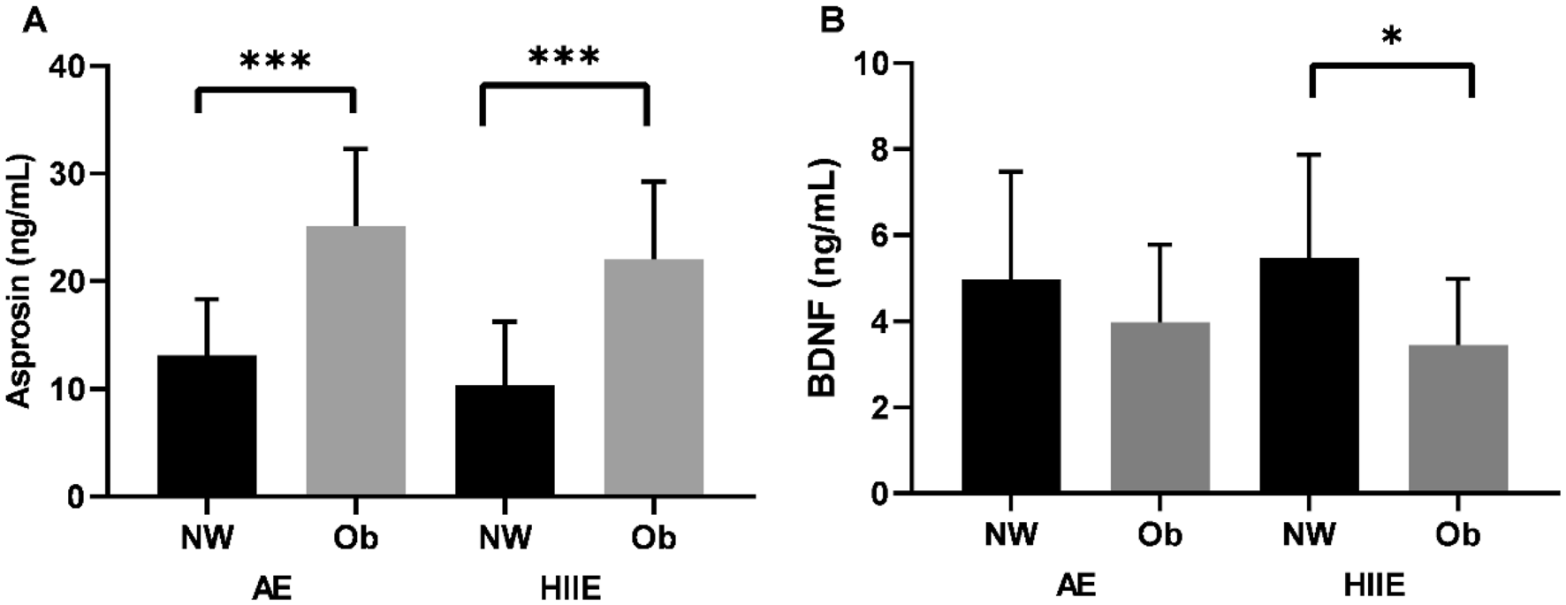
Comparison of asprosin and BDNF (basal) levels of NW and Ob groups before AE and HIIE. *p < 0.05, ***p < .001, NW: Normal weight; Ob: Obese, AE: Aerobic Exercise; HIIE: High Intensity Interval Exercise.

As shown in Fig. 2A, the basal level of serum asprosin was statistically significantly higher in the Ob group compared to the NW group [AE; t_(18)_ = − 4.315, NW *p* < 0.001, Cohen’s d: − 1.93 (− 2.98;− 0.83, 95% CI, large effect); HIIE; t_(18)_ = − 3.943, *p* < 0.001, Cohen’s d: − 1.76 (− 2.70;− 0.70, 95% CI), large effect].

In Fig. 2B, the basal serum BDNF measured before the HIIE was statistically significantly lower in the Ob group than in the NW group [t_(18)_ = 2.261, *p* = 0.018, Cohen’s d: 1.01 (0.06;1.9, 95% CI), moderate effect]. Also, serum BDNF measured before AE did not differ statistically between the groups [t_(18)_ = 1.009, *p* = 0.163, Cohen’s d: 0.45 (− 0.44;1.3, 95% CI, small effect].

**Figure 3:**
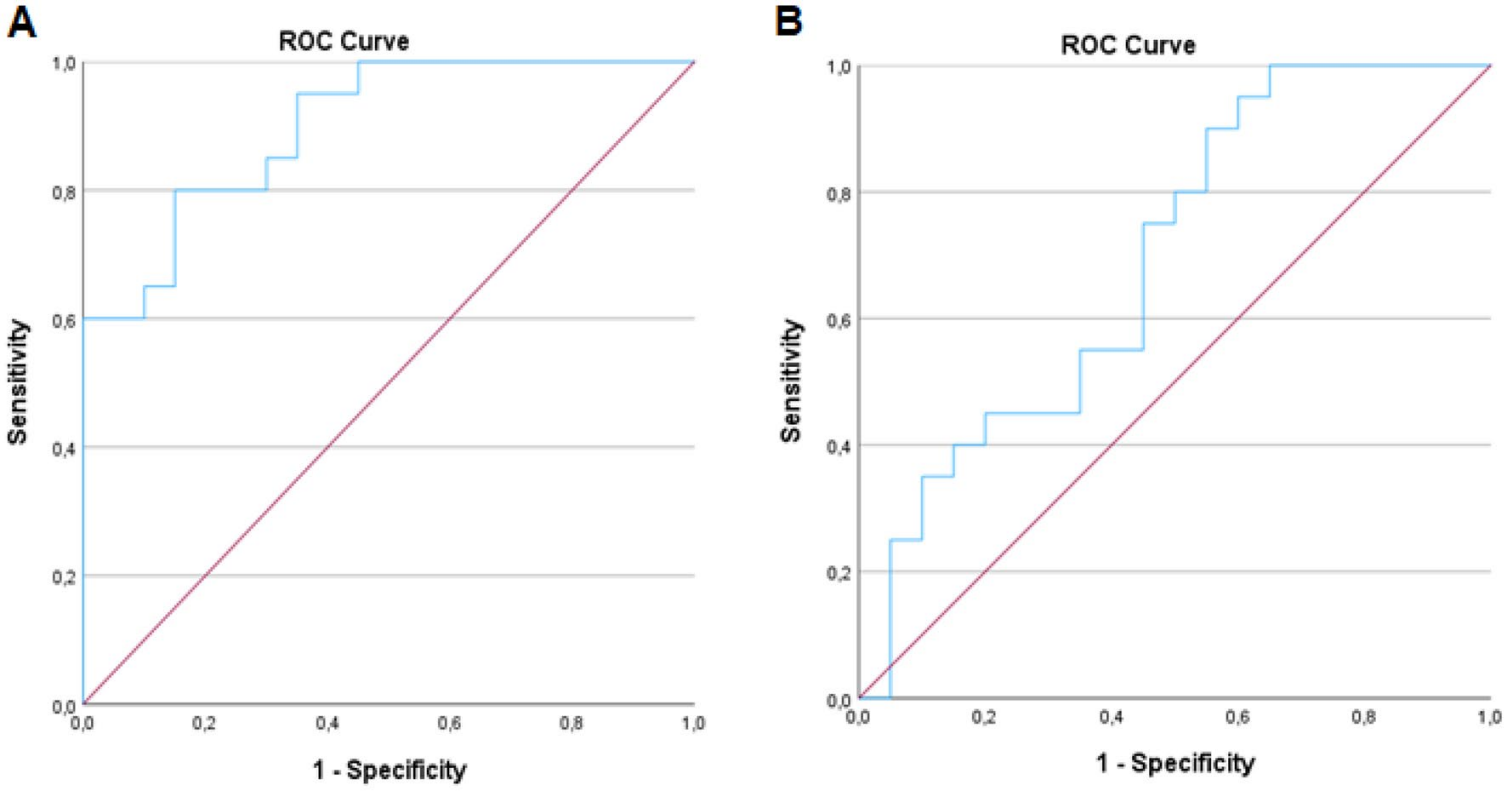
Receiver Operating Characteristic (ROC) Curve Analysis of serum asprosin (**A**) and BDNF (**B**).

Receiver operating characteristic analysis results showed cutoff values for serum levels of asprosin and BDNF in Table 2 and Fig. 3. The serum levels of asprosin were found to have an area under the curve (AUC) of 90.0% and a cutoff value of 17.8 ng/mL, with 80.0% sensitivity and 85.0% specificity. Moreover, the serum levels of BDNF were found to have an area under the curve (AUC) of 69.2% and a cutoff value of 6.36 ng/mL, with 95.0% sensitivity and 40.0% specificity.

**Table 2:** ROC analysis of serum asprosin and BDNF levels.

Variables	AUC (95% CI)	Cut off	*p*	Sensitivity	Specificity
Serum Asprosin	0.900 (0.809–0.991)	17.8	0.000	0.800	0.850
Serum BDNF	0.692 (0.527–0.858)	6.36	0.037	0.950	0.400

Significant differences (*p* < 0.05). Abbreviations: AUC, area under the curve; ROC, Receiver Operating Characteristic.

Considering these results, the present study demonstrated that serum asprosin level compared to serum BDNF had an important predictive and diagnostic value in obesity.

**Figure 4:**
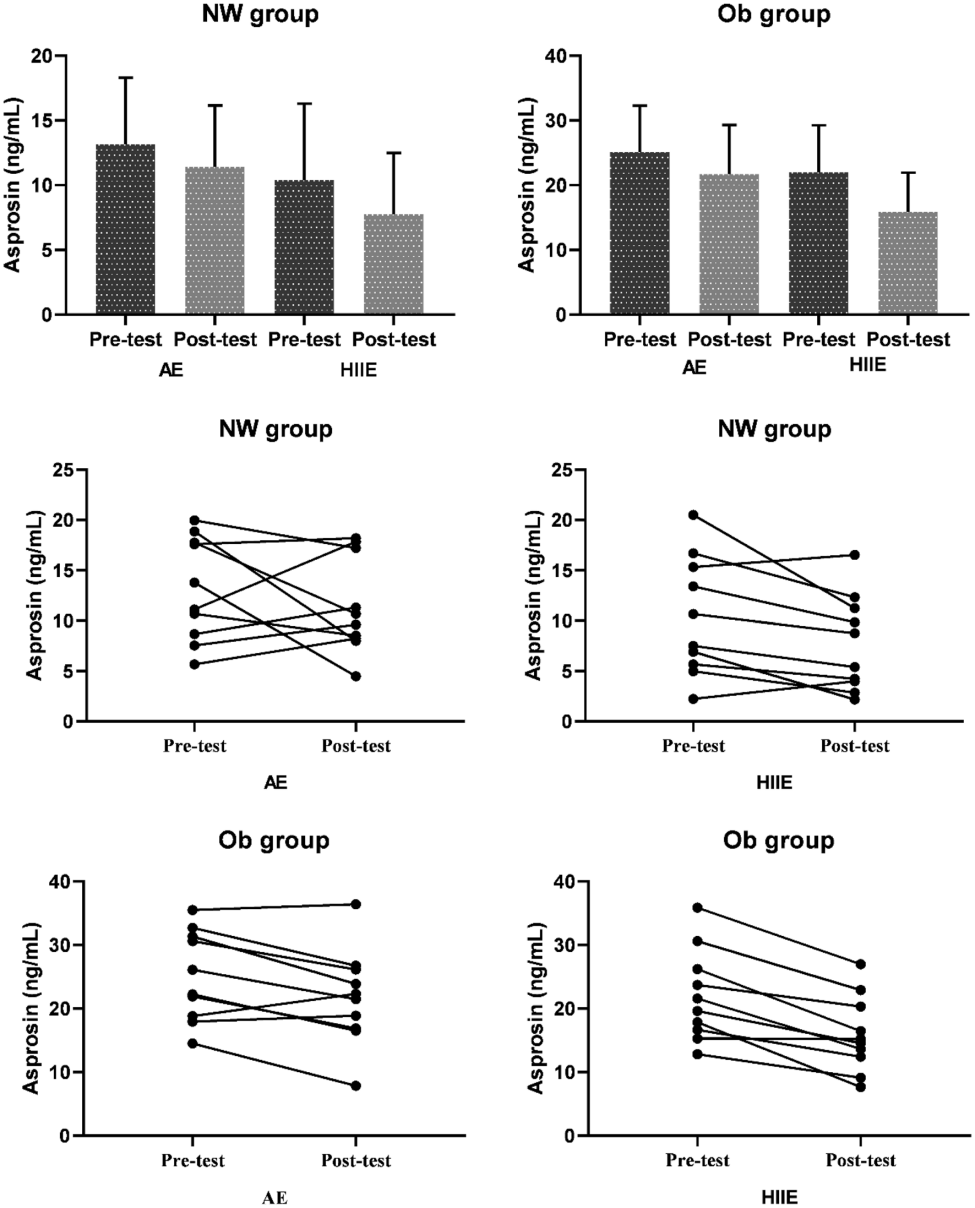
Serum asprosin levels (bar charts and scatter plots) of NW and Ob groups before and after AE and HIIE. NW: Normal weight; Ob: Obese, AE: Aerobic Exercise; HIIE: High Intensity Interval Exercise.

In Fig. 4, statistically significant difference was found in the comparison of the mean serum asprosin value before and after exercise in the NW group, regardless of exercise intensity (AE and HIIE) [F_(1,18)_ = 4.512, *p* = 0.048, η^2^p: 0.200]. According to the Bonferroni test results, it was observed that the serum asprosin level of the NW group was significantly decreased after both AE and HIIE [NW group: standardized effect size: 0.41 (− 0.00; 0.83, 95% CI, small effect]. For the Ob group it was observed that serum asprosin level decreased significantly after both AE and HIIE, independently of exercise intensity [F(1.18) = 36,063, *p* < 0.001, η^2^p: 0.667, standardized effect size: 0.66 (0.33; 0.98, 95% CI, moderate effect].

In both groups, it was determined that the interaction of Exercise intensity*Test Time (pre-test_post-test) did not have a statistically significant effect on serum asprosin level [NW: F_(1,18)_ = 0.187, *p* = 0.670, η^2^p: 0.010; Ob: F_(1,18)_ = 2.754, *p* = 0.114, η^2^p: 0.133].

In addition, a statistically significant difference was found in the comparison of the mean value of serum asprosin level before and after both AE and HIIE, without any group distinction (NW and Ob) [AE: F_(1,18)_ = 5.700, *p* = 0.028, η^2^p: 0.241; HIIE: F_(1,18)_ = 36.863, *p* < 0.001, η^2^p: 0.672]. According to the Bonferroni test results; serum asprosin levels were significantly reduced after AE [standardized effect size: 0.31 (0.02; 0.59, 95% CI, small effect], and HIIE [standardized effect size: 0.47 (0.22; 0.71, 95% CI, small effect] in both groups.

For AE, the Group*Test Time interaction did not significantly affect serum asprosin level [F_(1,18)_ = 0.605, *p* = 0.447, η^2^p: 0.033].

For HIIE, it was observed that the Group*Test Time interaction significantly affected the serum asprosin level [F_(1,18)_ = 5.696, *p* = 0.028, η^2^p: 0.240]. According to the Bonferroni test, there was a significantly higher decrease in serum asprosin level in the Ob group compared to the NW group after HIIE [NW: standardized effect size: 0.46 (− 0.00; 0.93, 95% CI, small effect); Ob: standardized effect size: 0.86 (0.29; 1.42, 95% CI, moderate effect].

**Figure 5:**
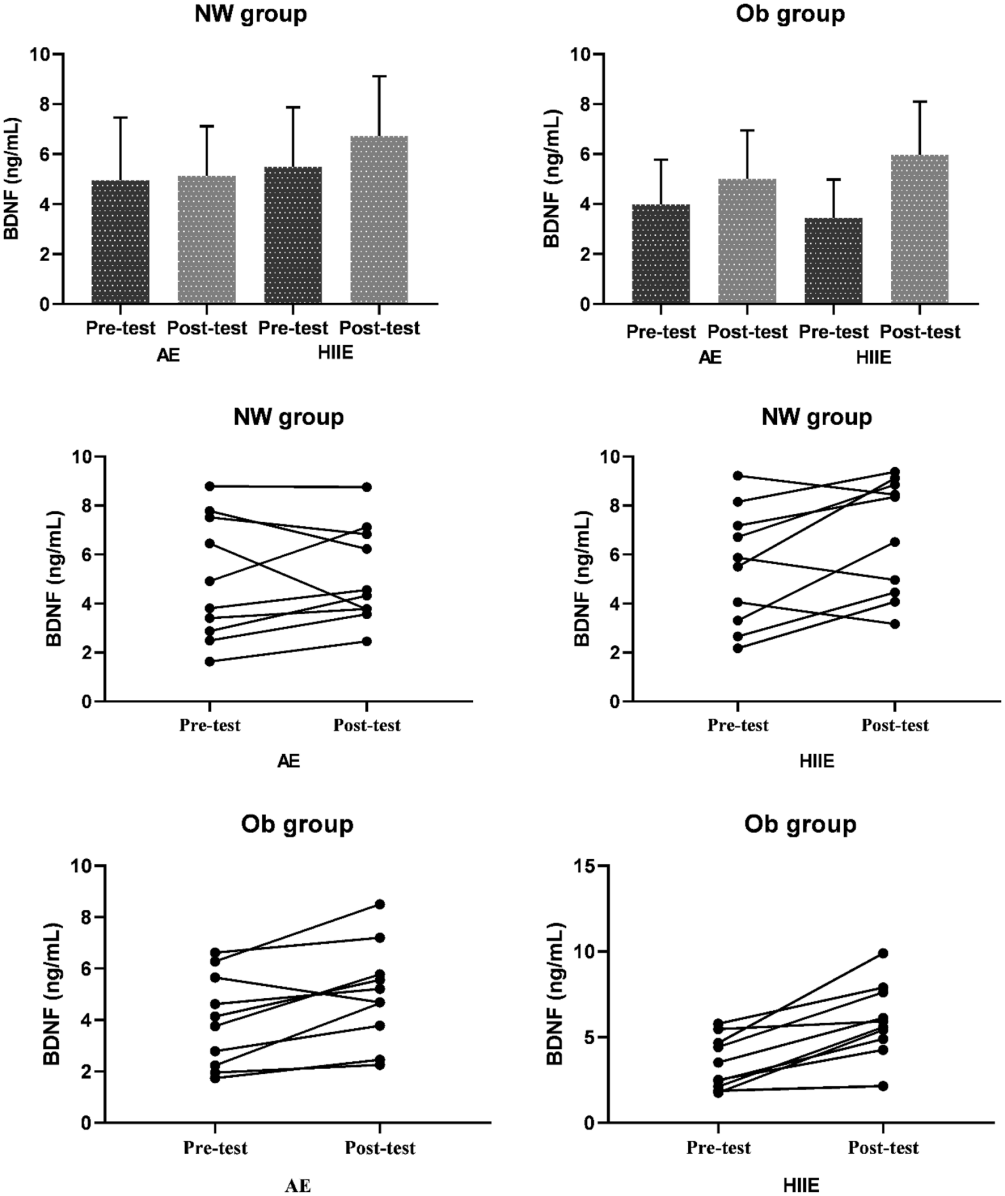
The changes (bar charts and scatter plots) in serum BDNF levels of NW and Ob groups before and after AE and HIIE. NW: Normal weight; Ob: Obese, AE: Aerobic Exercise; HIIE: High Intensity Interval Exercise.

As shown in Fig. 5, no statistically significant difference was found in the comparison of mean serum BDNF values before and after exercise for the NW group, regardless of exercise intensity (AE, HIIE) [F_(1,18)_ = 4.164, *p* = 0.056, η^2^p: 0.188]. In addition, it was observed that exercise intensity*Test Time did not have a significant effect on serum BDNF [F(1.18) = 2.401, *p* = 0.139, η^2^p: 0.118].

For the Ob group, it was determined that the serum BDNF level increased significantly post-exercise compared to pre-exercise, regardless of exercise intensity (AE, HIIE) [F_(1,18)_ = 38.269, *p* < 0.001, η^2^p: 0.680, standardized effect size: − 0.93 (− 1.4; − 0.45, 95% CI, moderate effect]. Moreover, it was seen that the Exercise intensity*Test Time had a statistically significant effect on serum BDNF [F_(1,18)_ = 6.765, *p* = 0.018, η^2^p: 0.273].

According to this result, more increases were found in serum BDNF levels after HIIE compared to AE in Ob group [AE: standardized effect size: − 0.54 (− 0.1.0; − 0.06, 95% CI, small effect; HIIE: standardized effect size: − 0.1.27 (− 2.1; − 0.40, 95% Cl, large effect].

For AE, a statistically significant difference was found when comparing the mean value of serum BDNF level before and after exercise, regardless of groups [F(1.18) = 4.499, *p* = 0.048, η^2^p: 0.200, standardized effect size: − 0.28 (− 0.59; 0.02, 95% CI, small effect]. It was found that the Group*Test Time interaction had no statistically significant effect on serum BDNF [F(1.18) = 2.300, *p* = 0.147, η^2^p: 0.113].

For HIIE, post-exercise serum BDNF levels were significantly higher than pre-exercise, without group discrimination (NW and Ob). [F_(1,18)_ = 28.783, *p* < 0.001, η^2^p: 0.615, standardized effect size: − 0.84 (− 1.3; − 0.39, 95% CI, moderate effect]. The Group*Test Time interaction did not significantly affect serum BDNF [F_(1,18)_ = 3.273, *p* = 0.087, η^2^p: 0.154]. Although it did not show a significant effect, serum BDNF level increased more in the Ob group than in the NW group [NW: standardized effect size: − 0.52 (− 0.1.0; 0.04, 95% CI, small effect; Ob: standardized effect size: − 1.2 (− 2.1); − 0.40, 95% CI, large effect].

## Discussion

The present study aimed to analyze the acute effects of moderate-intensity aerobic and high-intensity interval exercise protocols on asprosin and BDNF levels in inactive normal weight and obese individuals. Our results showed that, at rest, asprosin was significantly higher in the Ob individuals compared to the NW, in contrast to BDNF levels, which were lower in the Ob group. In both asprosin and BDNF, physical exercise seems to exert more influence on Ob than in NW group. Moreover, the higher intensity of physical exercises seems to implement greater results on asprosin (decline) and BDNF (increase), especially on BDNF levels (large vs small effects on HIIE and AE training, respectively). Those results should be considered when planning training programs as it could contribute to the treatment of obesity and several neuro-psychological diseases (BDNF, especially brain health) and glucose metabolism disorders (asprosin) that accompany obesity due to appetite signal disorder^61–65^.

Asprosin level was considered a potential independent risk factor in obesity etiopathogenesis^37^. Considering ROC analysis results, the present study demonstrated that serum asprosin level compared to serum BDNF had an important predictive and diagnostic value in obesity. Cantay et al. (2022) found that serum levels of asprosin had an area under the curve (AUC) value of 53.0% and a cutoff value of 6.97 ng/mL, with 51.4% sensitivity and 48.6% specificity^37^. Another study performed the patients with obesity showed that serum levels of asprosin had an AUC value of 77.0% and a cutoff value of 144 ng/mL, with 71% sensitivity and 71% specificity^66^. In our study, the serum levels of asprosin were found to have an AUC of 90.0% and a cutoff value of 17.8 ng/mL, with 80.0% sensitivity and 85.0% specificity. Considering the studies mentioned above, different results may be related to the characteristics of the participants, time of collection of blood samples from participants, the brand of the kit used, and the standard curve range ranges (0.5–100 ng/mL in the present study). Increasing the number of studies showing cut-off points for obesity for both asprosin and BDNF may contribute to understanding obesity pathology.

Asprosin has been considered an important hormone in appetite regulation, since it was observed that it crosses the blood–brain barrier, directly affecting appetite stimulation^67^. Considering that, studies has been reported that asprosin presented higher levels in obese than in normal weight subjects^39,66,68^. Hence, it has been discovered that asprosin increase approximately twofold to threefold in obese I and II classes and increased approximately fourfold in obese class III^69,70^. Thus, those greater levels of asprosin, might create a feeling of hunger, and triggers more food intake, and as a result, obesity might develop due to an increase in body weight and body fat percentage in these individuals^39^. Therefore, asprosin has been considered a potential pharmacological tool in treating obesity^71^. The present study is on those results, since Ob group presented greater asprosin levels when compared to NW subjects.

When implementing physical exercise, it was observed that asprosin declined. However, this effect was more pronounced in Ob subjects, since in all analysis (Ob together with NW, and in AE and HIIE separately) it always showed decreases. In NW, when analyzing the physical exercise intensities (AE and HIIE) separately, and also NW together with Ob group, it was noticed significantly decreases, in contrast to NW alone with the two intensities of physical exercise. The higher the basal asprosin values, the greater its reduction with induced physical exercise could be. Although it is unclear which mechanisms cause decline in appetite after acute exercise, it was suggested that certain mechanisms led to decline in appetite after exercise^72^. Therefore, as Wang et al. (2019) suggested for asprosin, physical exercise may have more complex functions than just influencing appetite control and glucose regulation, with a greater effect when applied integrated^66^. Physical exercise has been considered the best non-pharmacological treatment for obesity, as it results in negative energy balance and weight loss^73–75^.

Studies has been suggested an association between obesity and impaired cognitive functions^76^. In fact, it is known that adipose tissue is a store of fat reserves and an extremely active endocrine organ^77^. Considering that, researchers has been studied the BDNF concentrations on obese people. BDNF is a member of the nerve growth factor related family expressed in the central and peripheral nervous system^78^, which plays an important role in several brain plasticity aspects, regulating metabolic functions (fat oxidation and glucose uptake), contributing to cardiovascular improvement, and reducing the risk for neurodegenerative diseases^79^. Recent literature indicates that this neurotrophin modulates cognition, neuroplasticity, and angiogenesis and strengthens neural connectivity, being these activities are crucial for the development of learning and memory and contribute to better academic performance and brain health^9,80^. The results of the present study, seem to strengthen the assumption above mentioned, since lower basal values of BDNF on Ob when comparing to NW group were registered.

Furthermore, in the present study, as mentioned above, the basal level of serum BDNF was found to be statistically lower in the Ob group compared to the NW group. Consistent with our study, previous studies showed that BDNF was lower in Ob individuals compared to NW individuals^30,81^. Contrary to our study, the latest meta-analysis, including 10 studies, reported that the baseline BDNF levels of Ob and NW individuals did not differ significantly^27^. It was shown that the reason for the insignificance of BDNF levels between Ob and NW individuals might be due to some biases in the data evaluation (e.g. sample group, sampling, handling and storage procedures), which might affect BDNF measurements, and led to difficult interpretation of the results^27^. Therefore, there is still a need to standardise procedures and better quality and controlled research to achieve robust, cost-effective and reliable results. In addition, identifying the mechanistic consequences of BDNF/TrkB signaling in resolving the relationship between obesity and BDNF could be an important step towards developing new treatment strategies for obesity and its associated medical complications^82,83^. Finally, more clinical studies are needed to explain the relationship and underlying mechanisms between obesity and BDNF.

Several studies have been reported that acute exercise increases circulating levels of BDNF^9,21,84–87^. Hence, an inverse association between the peripheral BDNF concentration and body mass index (BMI) in children and adults has been reported^88^. Broadly speaking exercise stimulates the release of neurotransmitters and neurotrophins in an activity-dependent manner, which acutely potentiates neural function and induces a cascade of events that promote structural and functional plasticity in brain^89–91^. However, the type of physical exercise, especially its intensity, still equivocal. On the one hand, one systematic review suggests BDNF increases intensity-dependently^92^.

On the other hand, others report that exercise duration drives this response^93^. Literature claims the same increasing circulating BDNF with acute aerobic and resistance exercise modalities^93^. Nevertheless, as in a study with rats^94^, the present study seems to suggest that physical exercise intensity matters, with higher intensities (HIIE) presenting a large effect, instead of a small effect registered by AE intensity. Our results were supported by a previous study conducted on Ob individuals which indicated that higher increases were detected in the Ob group compared to the NW group after acute HIIE protocol compared to acute moderate-intensity exercise^33^. Subsequently, it was noted that acute HIIE could be an effective strategy for up-regulating BDNF expression in an Ob population, independent of increased lactate and cortisol levels. In the literature, the greater effect of HIIE protocols on BDNF than AE is based on certain physiological mechanisms. First, a previous study suggested that there might be higher platelet aggregation and activation (exercise-induced thrombocystosis) after HIIE compared to AE protocol^95^. Since most of the peripheral BDNF is stored in platelets, platelets containing BDNF led to elevated serum BDNF concentrations due to increased spleen contractions with exercise^96^. Second, acute exercise causes an augment in exercise insulin-like growth factor-1. It was stated that this may trigger an increase in BNDF level in the hippocampus for its modulation of exercise-induced synaptic and cognitive plasticity^97^. Lastly, in a previous study, it was reported that increased BDNF elevated the expression of glucose transporter 4 (GLUT4) in skeletal muscle^98^.

Moreover, it was asserted that this might significantly impact substrate utilization or glucose metabolism, and may improve the modulation of obesity-related metabolic disorder^99^. Lastly, in a previous study, it was stated that the BDNF leves showed a circadian rhythm, and the highest BDNF levels were detected in the morning (around 08.00 a.m.), and also this hormone decreased throughout the day^100^. In our study, both AE and HIIE were performed between 08.00 and 10.00 a.m in the morning. Therefore, the circadian rhythm may also have played a role in the increase in BDNF levels after exercise. Based on these results, it seems important that Ob individuals prefer especially morning exercises in triggering the increase of BDNF level, taking into account the circadian rhythm factor of BDNF. This may contribute to the regulation of hormones associated with appetite regulation and the prevention of neuropsychological diseases (depression, bad mood etc.) accompanying obesity,

The present study includes some limitations. First, the participants' food consumption records and hydration levels were not followed during acute exercise protocols. With the 24-h retrospective food record, the participants' food consumption records could be kept for two days on weekdays and one day on weekends for a total of 3 days, and their energy intake could be calculated. Second, our study was carried out only on male participants. This makes it difficult to generalize the results, especially for women. Third, biochemical and hematological parameters were not examined together with the hormones we discussed in present study. This may also be a limitation. The present study or studies with different experimental designs can be performed in both male and female populations in different age ranges or individuals with various metabolic diseases (obesity, hypertension, type 2 diabetes, insulin resistance etc.), taking into account the daily energy intake (especially by applying standard food before the exercises, in a more controlled environment in the laboratory) of the participants and examining some hematological and biochemical parameters, and different hormones associated with obesity.

## Conclusion

The present study followed the literature showing that at rest, Ob individuals register higher asprosin and lower BDNF values than NW individuals. Physical exercise seems to influence more Ob than NW group in both asprosin and BDNF, with the highest intensity of physical exercise implementing greater results on asprosin (decline) and BDNF (increase), especially on BDNF levels (large vs small effects on HIIE and AE training, respectively). Those insights seem to increase our knowledge to fight obesity, which is important to consider when planning training programs for the Ob population. Also, detecting the mechanisms between the obesity, hormones mentioned above, and acute-chronic exercises may contribute to the treatment of obesity and various neuro-psychological diseases (BDNF, especially brain health) and glucose metabolism disorders (asprosin) accompanying obesity due to appetite signal disorder.

## Data Availability

Data are available for research purposes upon reasonable request to the corresponding author.
